# The diagnostic utility of Immunoglobulin G (IgG) avidity in distinguishing between past and acute infection of West Nile Virus (WNV)

**DOI:** 10.1128/jcm.00952-25

**Published:** 2025-09-19

**Authors:** Yaniv Lustig, Vicki Indenbaum, Ravit Koren, Shiri Katz-Likvornik, Osnat Halpern, Ella Mendelson

**Affiliations:** 1 Central Virology Laboratory, Public Health Services, Ministry of Health, Sheba Medical Center 549141 , Tel Hashomer, Israel; 2 Gray Faculty of Medical and Health Sciences, Tel Aviv University 26745, Tel Aviv-Yafo, Israel; Mayo Clinic Minnesota, Rochester, Minnesota, USA

**Keywords:** West Nile Virus, WNV diagnosis, avidity, IgG antibodies, WNV infection

## Abstract

Infection with West Nile Virus (WNV) can cause severe disease; however, due to immunoglobulin M (IgM) persistence and short viremia, confirmatory diagnosis of acute WNV infection often requires two consecutive samples and a laborious neutralization assay. Immunoglobulin G (IgG) avidity assay is being used to differentiate between acute and past infection of several viral diseases but not of WNV. To test whether IgG avidity can be utilized for WNV diagnosis, serum samples from 103 past and 84 acute WNV-infected persons diagnosed in our laboratory according to WHO guidelines were subjected to IgG avidity. ROC curve analysis was applied to measure the diagnostic accuracy of WNV IgG avidity and establish the optimal cut-off. Mean avidity and geometric mean neutralization titers (GMTs) from acute and past infected cases were 26% (95% CI: 21–30) and 183 (95% CI: 144–235) and 63% (95% CI: 59–67) and 110 (95% CI: 82–147), respectively. Optimal avidity cut-off level to distinguish between acute and past infection was 35% with sensitivity, specificity, PPV, and NPV of 81% (95% CI: 72–88), 88% (95% CI: 81–93), 55% (95% CI: 42–67), and 96% (95% CI: 94–98), respectively. Our results suggest that IgG avidity can rule out acute WNV infections in patients with positive WNV IgM and IgG antibodies and replace the neutralization assay as a confirmatory assay in most cases. By using an avidity assay as part of the routine diagnostic protocol of WNV, the diagnosis of WNV will be simplified, rapid, and would substantially reduce the workload and diagnostic difficulty of this flaviviral infection.

**IMPORTANCE:**

Diagnosing West Nile Virus (WNV) infection typically requires two serum samples collected weeks apart and a labor-intensive confirmatory test using live virus. Here, we evaluated immunoglobulin G (IgG) avidity assay, which measures the binding strength between WNV antigen and IgG antibodies, as a simpler diagnostic approach using only one sample. Our findings showed that IgG avidity reliably identifies past WNV infections but is less effective in confirming the acute phase. Crucially, this method can rule out acute infections and allow for a rapid and definitive diagnosis in most cases. IgG avidity assays offer a streamlined alternative, reducing the need for complex tests and prolonged timelines.

## INTRODUCTION

West Nile Virus (WNV) is one of the most widely circulating encephalitic orthoflaviviruses. Infection with WNV is mostly asymptomatic. However, in approximately 20% it results in West Nile Fever (WNF) and in less than 1% in acute West Nile Neuroinvasive Disease (1). Diagnosis of WNV is challenging. Due to the low level of viremia at the time of symptom onset and the rapid decline thereafter, WNV RNA is rarely detected in serum, plasma, or cerebrospinal fluid (CSF) (2), although urine and whole blood sample types were found to have a longer window of detection (2, 3). Therefore, laboratory diagnosis of WNV infection is primarily based on the identification of specific immunoglobulin M (IgM) and immunoglobulin G (IgG) antibodies in serum or CSF (4, 5). Since IgM antibodies for WNV can persist for months to years after infection (6–9) and due to possible cross-reactivity with other orthoflaviviruses, the WHO, CDC, and ECDC guidelines postulate that positive IgM and IgG antibodies in serum are only suggestive of WNV acute infection and neutralization assay is required for confirmation (https://www.ecdc.europa.eu/en/west-nile-fever/facts). Neutralization can confirm the antibodies' specificity for WNV and distinguish between acute and past infection.

A critical priority is to develop simple, fast, and accurate assays that will distinguish between acute and past WNV infection for sera with positive IgM and IgG antibodies. Such an assay will allow confirmation of WNV diagnosis in a single serum sample. One assay that may aid in WNV diagnosis is IgG avidity, which measures the affinity between serum IgG antibodies and viral antigens (10). Current protocols suggest that low affinity (<40%) is suggestive of acute infection, while high affinity (>60%) is suggestive of a past infection (11), although it is important to note that low affinity may be associated with cross-reacting antibodies as well. Several studies have previously evaluated the clinical significance of IgG avidity testing for CMV (12, 13), toxoplasmosis (14), rubella (15, 16), varicella zoster virus (17), and measles (18, 19). Interestingly, the cut-offs for low and high IgG avidity representing acute and past infection, respectively, vary between the specific viral infections. However, despite the fact that a few studies tried to examine the use of IgG avidity in distinguishing between acute and past WNV infection (20–22), it is currently not recommended for diagnostic confirmation of WNV infection.

In this study, we examined IgG avidity among confirmed acute and convalescent WNV-infected individuals and determined the performance of the test and its utility in distinguishing between acute and past WNV infections.

## MATERIALS AND METHODS

A diagnostic validation study was performed on serum samples tested for WNV infection at the Israeli national center for zoonotic viruses. Samples were defined as acute or past WNV infection based on the following characteristics:

A WNV acute infection case was defined as confirmed if laboratory tests demonstrated at least one of the following four results: (i) isolation of WNV from serum, plasma, or CSF; (ii) detection of WNV RNA by Real-Time (qRT)–polymerase chain reaction (PCR) in serum, plasma, or CSF; (iii) presence of specific IgM antibodies in the CSF; and (iv) detection of WNV IgM in the first sample with seroconversion to IgG in a convalescent sample or no WNV IgM detection in the first sample with detection of WNV IgM in the convalescent sample or detection of WNV IgM and IgG in both samples with confirmation of at least a fourfold increase in the convalescent sample by a neutralization assay (2).

A case was defined as WNV past infection if laboratory tests were either positive for WNV IgG and negative for IgM or positive in two consecutive samples for both IgM and IgG but demonstrated less than a fourfold induction in titer in a WNV neutralization assay.

All acute and past infection samples were tested using the IgG avidity assay, and the relative avidity index (RAI) was determined. Conditions for IgG avidity were adopted from a previous study on WNV IgG avidity (21). Acute or past WNV infections were interpreted based on the RAI results. Sensitivity and specificity were calculated by comparing the true results (as diagnosed based on WHO criteria) of acute or past WNV infection for each sample with avidity. The true positive rate (Sensitivity) was plotted as a function of the false positive rate (100 − Specificity) in a ROC curve for different cut-off points of the IgG avidity assay. Each point on the ROC curve was represented with a sensitivity/specificity pair corresponding to a particular decision threshold. Based on this data, the optimal criterion with the highest specificity and sensitivity to distinguish between acute and past WNV infection was determined.

### Enzyme-linked immunosorbent assay (ELISA) for detection of WNV antibodies and avidity

Serum samples were tested for avidity using a prototype ELISA (IgG DxSelect ELISA kits by Focus Diagnostics Inc., Cypress, CA, USA). Briefly, 100 µL of serum diluted 1:100 in assay buffer was added to each of two wells coated with WNV. After incubation for 60 min at 25°C, duplicate wells were exposed to either 6 M urea solution or to phosphate buffer for 10 min. After washing, wells were incubated with peroxidase-labeled anti-human IgG for 30 min at 25°C, substrate was added, and the reactions were stopped after 10 min by the addition of 100 µL stop solution per well. The reactions were read immediately at a wavelength of 450 nm using a reference wavelength of 650 nm. RAI was calculated for each specimen and expressed as the percentage of reactivity remaining in the urea-treated well. The laboratory assay was performed in a blinded manner at the Israeli National Center for Zoonotic Viruses at the Central Virology Laboratory (CVL).

### Neutralization assay

All samples were tested by a micro-neutralization assay (23). Briefly, 100 median tissue culture infectious dose (TCID50) of WNV (lineage 1, isolated in Israel) was incubated with inactivated (56°C for 30 min) ELISA IgG-positive sera diluted 1:10 to 1:1,280 in 96-well plates for 60 min at 37°C. Vero E-6 cells were added to each well and incubated for 6 days. Following Gentian violet staining (1%), which stained and fixed the cell culture layer, the neutralizing dilution of each serum sample was determined by identifying the well with the highest serum dilution without observable cytopathic effect. A dilution equal to 1:10 or above was considered neutralizing.

### Data analysis

Microsoft Excel software was used to manage the data. GraphPad Prism 9.1.0 software was used to calculate the sensitivity, specificity, positive predictive value (PPV), and negative predictive value (NPV) of the IgG avidity and express them with 95% confidence interval (CI) as well as ROC curve analysis to determine the potential decisive threshold of the IgG avidity assay.

## RESULTS

**Fig 1:**
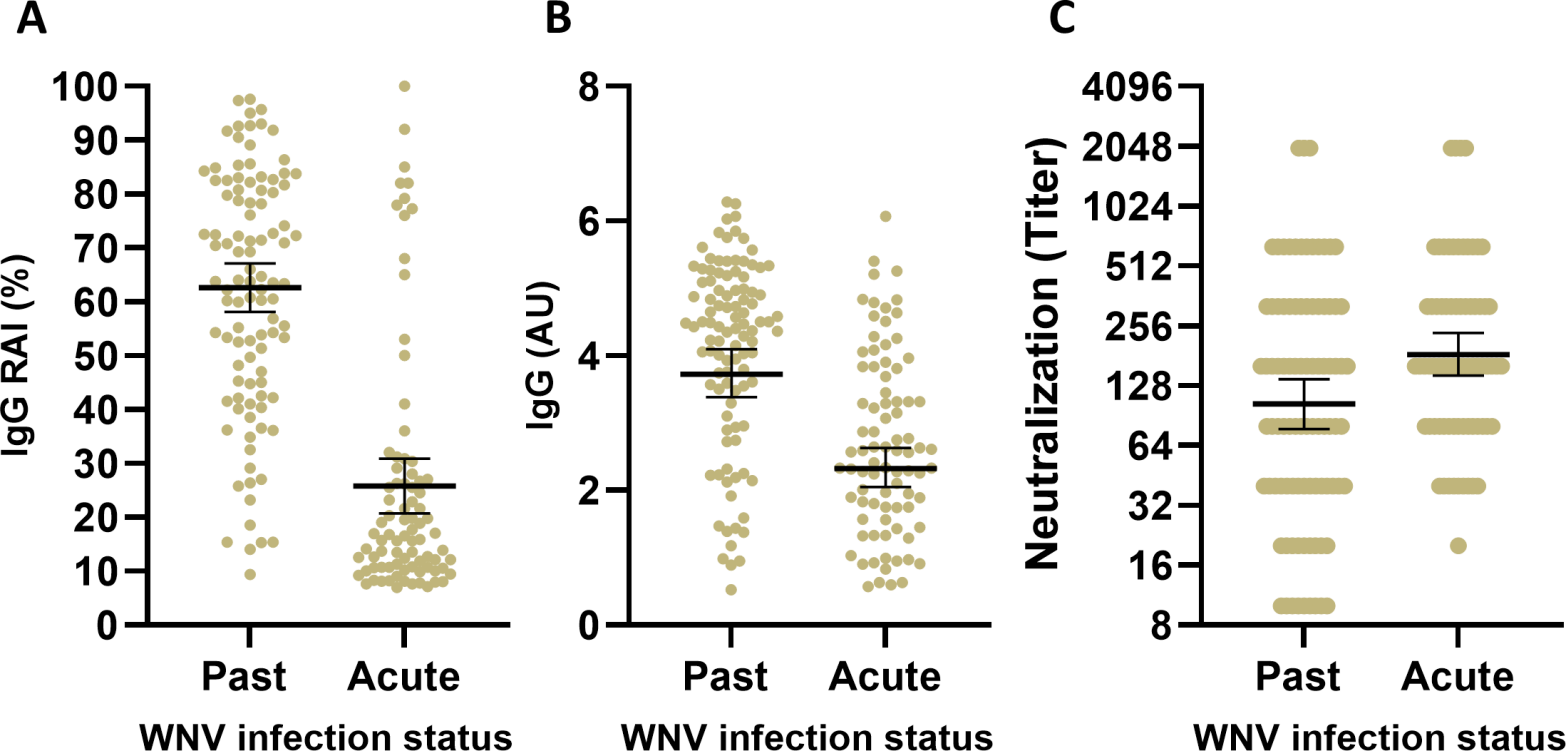
IgG avidity, IgG levels, and neutralization of acute and past WNV-infected individuals. Scatter plot analysis of relative avidity index (RAI) (**A**), IgG levels (**B**), and neutralization titers (**C**) in confirmed WNV acute and past-infected individuals. The black bars indicate mean (for avidity and IgG) or GMT (for neutralization) ±95% CI. Reciprocal neutralization titers are depicted.

Eighty-four serum samples from confirmed acute WNV infections and 103 serum samples from past WNV infections, obtained between 2015 and 2023, were tested for avidity and WNV neutralization. For the 84 samples from acute infection and 103 samples from past infection, RAI, IgG levels (AU) and neutralization titers ranged from 7% to 100% (mean = 26%; 95% CI: 21–31), 0.6–6.1 AU (mean = 2.3 AU; 95% CI: 2.1–2.6) and titers of 20–2,000 (geometric mean titer [GMT] = 183; 95% CI: 144–235) in the acute group and from 9% to 100% (mean = 63%; 95% CI: 59–67), 0.5–6.3 AU (mean = 3.7 AU; 95% CI: 3.4–4.1) and titers of 10–2,000 (GMT = 110 95% CI: 82-147) in the past infection group, respectively (Fig. 1).

As mentioned, several commercial assays as well as a study aimed to differentiate between early and late antibody response to WNV defined IgG avidity below 40% as acute infection and IgG avidity above 60% as past infection despite using different IgG avidity kits and reagents (6, 20–22). Therefore, we first evaluated the performance of the IgG avidity assay based on this 40–60% criteria. Sensitivity based on this criterion in identifying acute infections was 83% (95% CI: 74–90) with a specificity of 85% (95% CI: 77–90). The sensitivity to exclude acute infections was 60% (95% CI: 51–69) with a specificity of 88% (95% CI: 79–93). Taken together (Table 1), the sensitivity and specificity to detect acute infection based on avidity of below 40% and past infections based on avidity of above 60% are 87% (95% CI: 78–93) and 80% (95% CI: 70–88), respectively. It is important to note that in this scenario, avidity between 40% and 60% is considered intermediate and therefore could not be interpreted as past or acute infection.

**TABLE 1:** Diagnostic tests based on known criteria (40% acute, 60% past)

For <40% and >60%	True acute	True past	Total
**Avidity acute**	70	17	**87^*a*^**
**Intermediate**	3	25	**28**
**Avidity past**	11	61	**72**
**Total**	**84**	**103**	**187**

^*a*^ Bold values indicate total values that summarize the numerical data presented.

While sensitivity and specificity of the assay are important and describe its performance, the utility of avidity as a tool used to differentiate between acute and past WNV infection depends on the PPV and NPV of the assay (18). In order to determine the PPV and NPV of IgG avidity, we need to estimate the prevalence of WNV confirmed infection in our population, i.e/, the ratio of WNV confirmed infections diagnosed at the CVL among the total number of samples with a positive WNV IgG. First, we assessed the ratio of suspected WND among all IgG-positive samples. Between 2015 and 2023, serum samples from 5,164 cases were sent to the CVL for diagnosis of WNV disease. Of the 1,023 serum samples positive for WNV IgG antibodies, 248 samples (24%) were also positive for IgM.

Next, we assessed the ratio of confirmed acute WND from all suspected cases. Between 2015 and 2023, 558 patients were diagnosed with a probable case of WND from both CSF and serum. Three hundred and fifty-six (64%) were confirmed for WND according to the diagnostic criteria.

Overall, these data show that the ratio of confirmed WNV cases diagnosed in the CVL among the total IgG-positive samples is 15% (64% out of 24%). Accordingly, the PPV and NPV to detect acute WNV infection based on avidity of below 40% and past WNV infections based on avidity of above 60% are 43% (95% CI: 33–54) and 97% (95% CI: 95–98), respectively. This result suggests that an avidity result above 60%, in a given sample with positive IgM and IgG antibodies against WNV, is 97% indicative of past infection, while avidity of less than 40% is only 43% indicative of acute infection, and avidity between 40% and 60% is undetermined.

**Fig 2:**
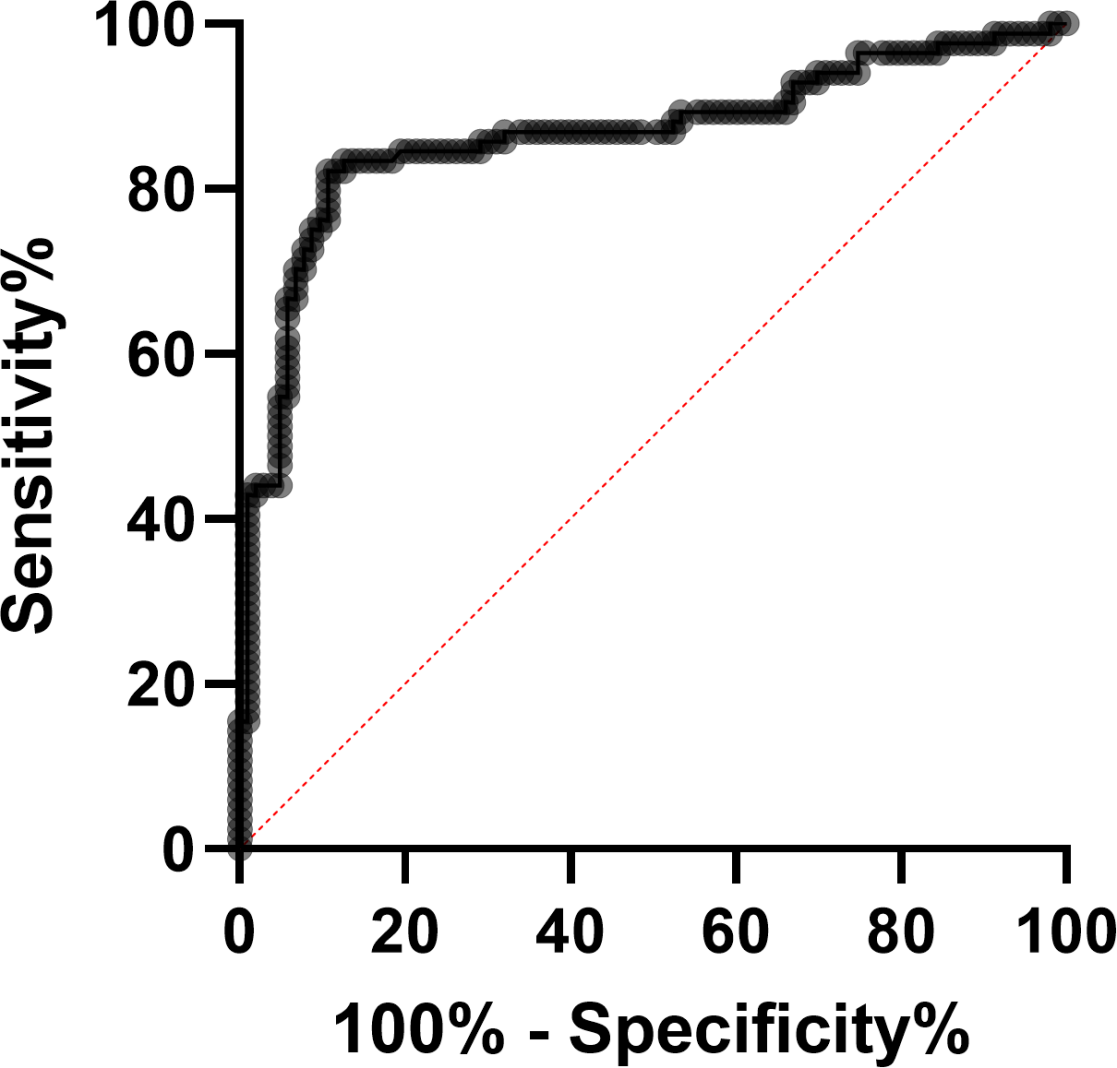
Performance of IgG avidity. AUC ROC analysis of WNV IgG avidity with IgG ELISA following the protocol depicted in the Materials and Methods section.

In order to predict the optimal cut-off for IgG avidity to diagnose acute and past WNV infections, we generated an AUC ROC curve. The AUC for the avidity index was 0.86 (95% CI: 0.81–0.92) (*P* < 0.001) (Fig. 2).

Next, we analyzed the sensitivity, specificity, PPV, and NPV of the avidity assay for a range of RAI cut-offs (Table 2). Since our aim is to increase the range of individuals with accurate diagnosis, the optimal RAI avidity score was determined to be 35 with sensitivity, specificity, PPV, and NPV of 81% (95% CI: 72–88), 88% (95% CI: 81–93), 55% (95% CI: 42–67), and 96% (95% CI: 94–98), respectively.

**TABLE 2:** Performance of the avidity assay

RAI	Sensitivity (%)	95% Cl	Specificity (%)	95% Cl	PPV (%)	95% Cl	NPV (%)	95% Cl	Accuracy (%)
<15	43	33–53%	98	94–100%	81	51–94%	91	89–92%	90
<25	67	57–76%	94	87–97%	65	47– 79%	94	92–96%	90
<35	81	72–88%	88	81–93%	55	42–67%	96	94–98%	90
<45	85	77–91%	77	69–84%	40	32–49%	97	95–98%	78
<55	88	79–93%	65	55–73%	30	25–36%	97	94–98%	68
<65	89	815–94%	48	39–57%	23	20–27%	96	93–98%	54
<75	91	83–95%	34	25–43%	19	17–22%	96	91–98%	42
<85	97	91–99%	15	9.95–23%	17	15–18%	96	88–99%	27

**Fig 3:**
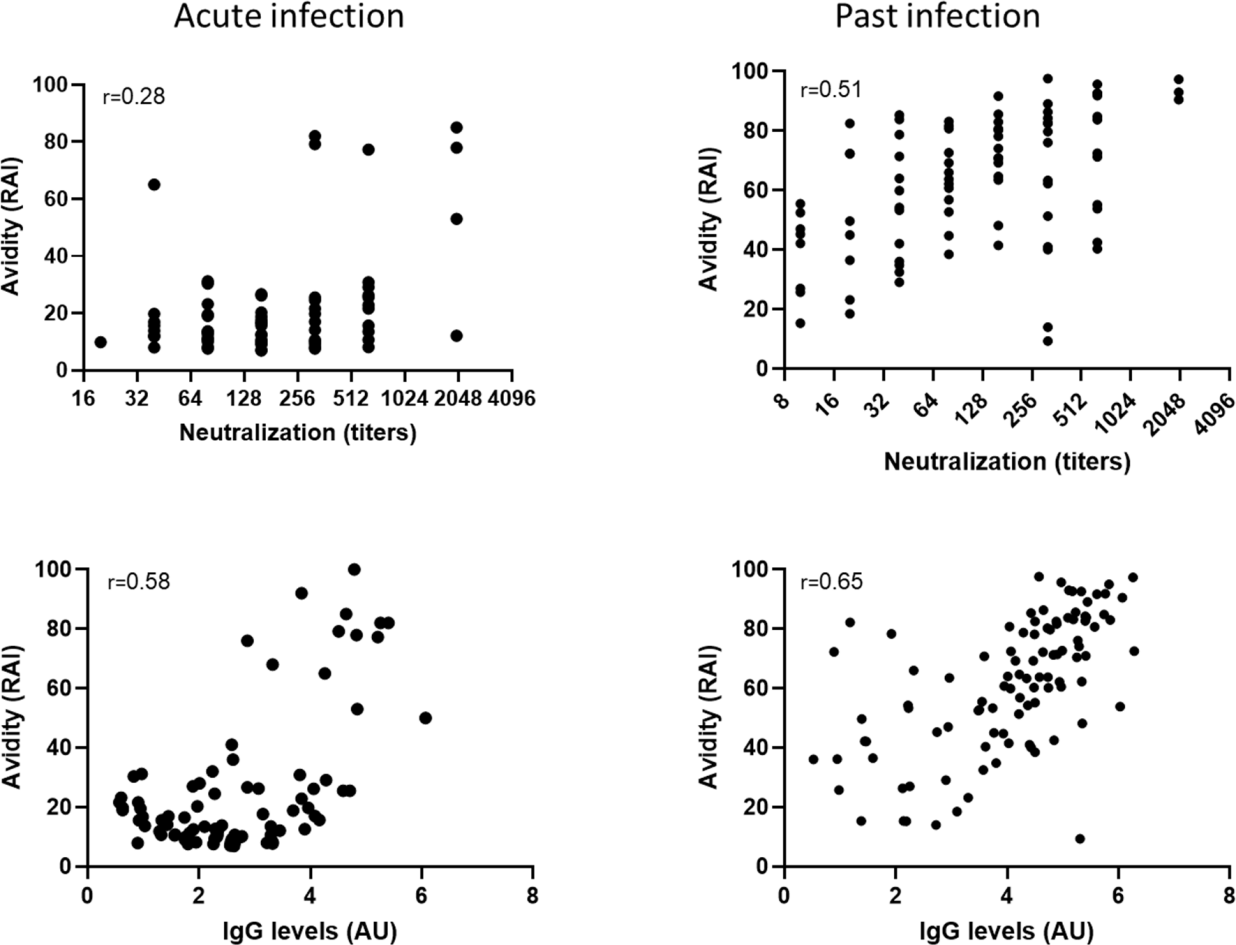
Correlation between avidity, IgG levels, and neutralizing titers. Correlation of avidity and neutralizing titers and avidity and IgG levels in samples obtained from acute (left panel) and past (right panel) WNV-infected individuals is presented. The correlation coefficient (R) is depicted for each correlation graph.

Since IgG antibodies measure quantity of antibodies, IgG avidity reflects the quality and affinity of these antibodies, and neutralization titers are a measure of both quality and quantity, we assessed the correlation between these parameters. For acute and past infections, the correlation coefficient between avidity and IgG levels was 0.58 (95% CI: 0.42–0.7) and 0.65 (95% CI: 0.52–0.75), respectively, and between avidity and neutralizing titers was 0.28 (95% CI: 0.04–0.48) and 0.51 (95% CI: 0.34–0.65), respectively (Fig. 3).

## DISCUSSION

Global warming, increased travel, and population expansion into new previously uninhabited areas have resulted in the emergence of zoonotic pathogens in countries all over the world (24). As such, WNV circulation is constantly spreading and currently encompasses most of North America, Europe, and the Middle East. In Israel, WNV outbreaks are frequent and in 2024 resulted in 934 cases, most with significant neurological symptoms. Due to the short and low-level viremia, serology is the primary tool for WNV diagnosis with the presence of IgM antibodies specific to WNV indicative of acute infection. Unfortunately, several studies and real-world experience revealed that WNV-specific IgM antibodies may persist for months and even years after initial infection (7–9), thus complicating the diagnosis of acute WNV infection based on the presence of IgM alone. In this study, we found that IgG avidity may be used to aid in WNV diagnosis. Using a well-defined cohort of acute and past-infected WNV cases, we were able to demonstrate that IgG avidity accurately identifies previously infected individuals but is less conclusive for detecting acute infection.

A few studies have previously evaluated IgG avidity levels following WNV infection (20–22) and demonstrated that IgG avidity levels are positively correlated with time since symptoms onset or positive RT-PCR for WNV RNA. However, due to the limited past-infection cohorts used, only a few of the samples tested for IgG avidity originated from older WNV convalescence samples or were clinical samples. Here, 187 samples, 103 convalescent and 84 acute samples from clinical samples were characterized based on the WNV diagnostic criteria and were confirmed as acute and convalescent real-world samples. Similarly to other studies (20–22), average avidity results from acute samples were low (26%; 95% CI: 21–30) and from past infected samples were high (63%; 95% CI: 59–67) (Fig. 1). However, the wide distribution of avidity levels, especially among samples characterized as past infection, suggested that further analysis is required in order to determine the diagnostic utility of IgG avidity.

While sensitivity and specificity assess the IgG avidity assay’s ability to differentiate between acute and past WNV infection, PPV and NPV assess the test’s effectiveness in a real-world setting considering the disease prevalence. Thus, PPV and NPV are the most important parameters needed to determine the utility of IgG avidity for WNV diagnosis. Based on real-world clinical diagnostic data tested at the CVL between 2015 and 2023, we estimated that the prevalence of WNV confirmed infection among all samples with IgG antibodies sent for WNV diagnosis is 15%. Consequently, the PPV and NPV of the test using IgG avidity criteria of 40%/60% were low (43%) and high (97%), respectively. Taking into account that all samples with avidity levels between 40% and 60% are considered intermediate and therefore could not be interpreted, this test could only determine past infection if avidity was >60%. Optimization of the avidity test performance using AUC and ROC analysis demonstrated that an avidity cut-off of 35 is optimal and results in PPV and NPV of 55% and 96%, respectively. While the PPV of 55% is higher than the PPV of 43% that was demonstrated for the 40/60 cut-off, it is still unsatisfactory in terms of determining acute infection. A significant advantage of the 35 cut-off, however, is that it increases the range of confirming past infection from >60 to >35 with no significant reduction in the NPV. Using this cut-off will substantially increase the number of samples that can be confirmed as past infection by avidity without the need to perform neutralization assay and ultimately will result in diagnosing acute infection quickly and with only a single sample.

Since both neutralization and IgG antibodies have been studied extensively for WNV diagnosis and the correlation between these two parameters is strong (25), it was important to also evaluate the correlation with avidity. Interestingly, we observed a moderate correlation between IgG levels and IgG avidity. This correlation may be influenced by the persistence of high-avidity IgG antibodies generated during the acute infection phase compared to low avidity IgG antibodies. Indeed, a similar correlation between IgG levels and avidity was found following infections with CMV (26), WNV (22), and SARS-CoV-2 (27). In addition, we found that the relationship between avidity, IgG antibodies, and neutralization differed between acute and past infected samples. In the past, infected samples, a similar moderate correlation between both IgG and avidity and neutralization and avidity was observed. However, in acute infected samples, a low correlation was detected between avidity and neutralization, while moderate correlation was observed between avidity and IgG (Fig. 3). A possible explanation could stem from the characteristics of the immune response where following WNV infection, a simultaneous gradual increase in WNV-specific IgG antibodies and in the affinity maturation process contributes to increased correlation between avidity and IgG. Neutralizing activity, which depends on both quantity and quality of antibodies, takes a longer time to mature, and as a result, the correlation to avidity is lower at this stage of the immune response. Maturation of this process, in the convalescent phase, leads to a better correlation of neutralizing titers and avidity.

Differentiation between acute and past WNV infection using IgG avidity can be affected by cross-reactivation with other circulating orthoflaviviruses. While known to cause outbreaks primarily in birds (28) and is rarely responsible for symptomatic infections in humans, Usutu virus (USUV), an orthoflavivirus with significant cross-reactivity to WNV, co-circulates with WNV in Europe (29) and also in Israel (30). A recent study by Berneck et al. (31) demonstrated that modified recombinant envelope proteins with selectively altered cross-reactive epitopes can differentiate between WNV- and USUV-induced antibody responses. Their approach, involving targeted mutations in conserved epitopes within domains II and III of the orthoflavivirus E protein, showed high specificity in distinguishing seroresponses in human serum samples. Integrating such differential antigen designs into avidity-based or confirmatory assays may further improve diagnostic accuracy in co-endemic areas and complement the utility of the IgG avidity assay presented in this study.

Our study has few limitations. First, because our cohort consisted of random samples coming to our lab, we could not determine the time of infection for all the past infection cohort and most of the acute infection cohort. However, since our confirmatory diagnosis is based on WHO and ECDC criteria, these results reflect current practice. Second, since all samples were obtained from residents of Israel, results of IgG avidity from samples obtained in other countries, where cross-reactivity with other orthoflaviviruses exists such as USUV and Dengue may be affected due to cross-reactivity.

### Conclusions

In conclusion, our results demonstrate that in a real-world setting, sera from acute and past WNV infection are highly correlated with low and high IgG avidity, respectively. With high NPV but low PPV, IgG avidity is best suited for ruling out acute infection rather than confirming it. Replacing neutralization with avidity as a confirmatory assay to exclude past WNV infection will subsequently enable rapid one-sample diagnosis in most cases.
